# Disparate ultrafast dynamics of itinerant and localized magnetic moments in gadolinium metal

**DOI:** 10.1038/ncomms9262

**Published:** 2015-09-10

**Authors:** B. Frietsch, J. Bowlan, R. Carley, M. Teichmann, S. Wienholdt, D. Hinzke, U. Nowak, K. Carva, P. M. Oppeneer, M. Weinelt

**Affiliations:** 1 Fachbereich Physik, Freie Universität Berlin, Arnimallee 14, 14195 Berlin, Germany; 2 Max-Born-Institut, Max-Born-Strasse 2a, 12489 Berlin, Germany; 3 Fachbereich Physik, Universität Konstanz, 78457 Konstanz, Germany; 4 Department of Physics and Astronomy, Uppsala University, PO Box 516, 75120 Uppsala, Sweden; 5 Charles University, Faculty of Mathematics and Physics, DCMP, Ke Karlovu 5, CZ-12116 Prague 2, Czech Republic

## Abstract

The Heisenberg–Dirac intra-atomic exchange coupling is responsible for the formation of the atomic spin moment and thus the strongest interaction in magnetism. Therefore, it is generally assumed that intra-atomic exchange leads to a quasi-instantaneous aligning process in the magnetic moment dynamics of spins in separate, on-site atomic orbitals. Following ultrashort optical excitation of gadolinium metal, we concurrently record in photoemission the 4f magnetic linear dichroism and 5d exchange splitting. Their dynamics differ by one order of magnitude, with decay constants of 14 versus 0.8 ps, respectively. Spin dynamics simulations based on an orbital-resolved Heisenberg Hamiltonian combined with first-principles calculations explain the particular dynamics of 5d and 4f spin moments well, and corroborate that the 5d exchange splitting traces closely the 5d spin-moment dynamics. Thus gadolinium shows disparate dynamics of the localized 4f and the itinerant 5d spin moments, demonstrating a breakdown of their intra-atomic exchange alignment on a picosecond timescale.

## Abstract (web summary)

Due the strength of the intra-atomic exchange interaction, it is generally assumed that alignment of spin moments in intra-atomic orbitals is quasi-instantaneous. Here, the authors demonstrate the breakdown of this relation between the 4f and 5d electrons in gadolinium following ultrashort optical excitation.

## Graphical abstract

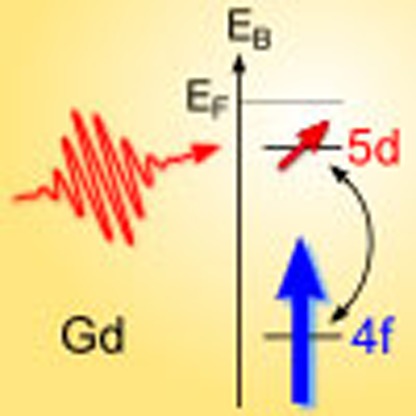

Ultrafast spectroscopic techniques provide new insights into correlated materials by exciting specific subsystems and following their subsequent relaxation. For magnetic systems, this involves probing non-equilibrium spin and charge dynamics that cannot be reached through thermodynamic pathways. The magnetism in metals is determined by the inter- and intra-atomic exchange interactions^1^. The former couples the spin moments on neighbouring atoms and leads to the long-range order, while the latter enforces the formation of the atomic spin moment. Inter-atomic exchange is typically much weaker than intra-atomic exchange, which is the strongest and thus potentially fastest force in magnetism. These magnetic interactions can be scrutinized on the shortest timescales by employing femtosecond laser pulses. Such investigations have recently been reported for metallic ferromagnets, half-metallic ferromagnets, dilute magnetic semiconductors, as well as correlated magnetic oxides^2^,^3^,^4^,^5^,^6^,^7^. The response of multicomponent magnetic materials to a short, fs-laser pulse has been examined in an element-specific manner using time-resolved core-level magneto-optical techniques in the soft-X-ray and extreme ultraviolet (XUV) regime^8^,^9^,^10^,^11^,^12^,^13^. These investigations showed that the inter-atomic exchange interaction can be overcome on short timescales. Distinct magnetic responses were seen for the iron and nickel atoms in permalloy on a sub-picosecond timescale^9^. Probing GdFeCo^8^, TbFe^14^, GdCo TbCo^13^, and GdTb alloys^15^, different dynamics of the Gd (or Tb) and Fe (or Co) spins were observed. The experiments on multi-sublattice ferrimagnets thereby provide evidence for transient inter-atomic decoupling on the timescale of a few picoseconds. The much stronger intra-atomic exchange could thus far not be examined, as this requires a dedicated technique to selectively probe spin-polarized electrons in different orbitals on the same atom.

Gadolinium metal is an ideal system to offer deep insight in the operation of intra-atomic exchange under non-equilibrium conditions. The localized Gd 4f electrons contribute most to the atomic moment with 7 *μ*_B_ per atom and spin-polarize the 5d valence electrons, which contribute an additional 0.55 *μ*_B_, where *μ*_B_ is the Bohr magneton. Because the 4f electrons of adjacent atoms have no spatial overlap, the neighbouring 4f moments align via intra-atomic (Hund's rule) exchange with the 5d electrons, which—combined with the inter-atomic exchange between 5d moments—leads to alignment of all moments (see Fig. 1a). The large intra-atomic exchange energy *J*_int_=130 meV, which corresponds to an exchange field of ∼4,000 Tesla, is the background upon which quasi-instantaneous alignment (*ħ*/*J*_int_∼6 fs; *ħ* is the reduced Planck constant) of on-site moments and, hence, identical spin dynamics have been assumed thus far.

Time- and angle-resolved photoemission spectroscopy (ARPES) with ultraviolet light pulses has developed into a sensitive tool to probe details of the photoexcited state on the ultrafast timescale^16^,^17^,^18^,^19^,^20^,^21^. Here we apply time-resolved ARPES using higher-order harmonic radiation to study within a single experiment the two spin subsystems of Gd metal, which are coupled via intra-atomic exchange. We show within a single time-resolved photoemission experiment that 5d exchange splitting and 4f magnetic linear dichroism (MLD) evolve upon 1.6-eV pulsed laser excitation on clearly different timescales with decay constants of 0.8 and 14 ps, respectively. We use first-principles calculations to derive the relation between the 5d exchange splitting and the 5d and 4f magnetic moments. The *ab initio* calculated intra- and inter-atomic exchange constants enter an orbital-resolved Heisenberg Hamiltonian used to simulate the spin dynamics. With the respective coupling of 5d and 4f spin systems to the electron and phonon heat baths (see Fig. 1a), the spin dynamics simulations explain the disparate dynamics of the 5d and 4f magnetic moments very well, despite the large intra-atomic exchange coupling of 130 meV.

## Results

### Time-resolved photoemission spectroscopy

**Figure 1:**
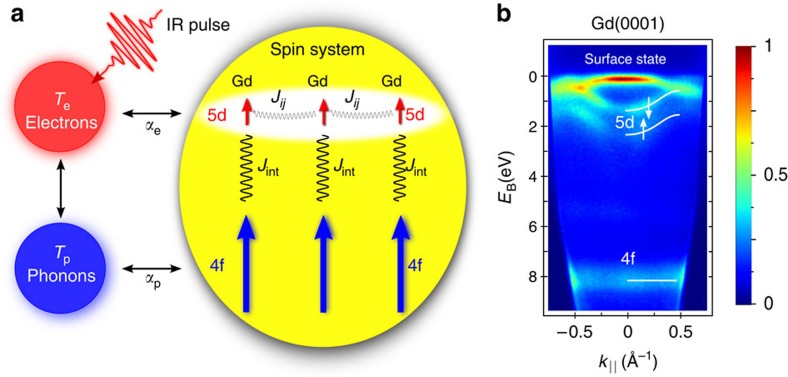
Couplings of the gadolinium spin system. (**a**) The interaction of the different heat baths in an extended three-temperature model. After infrared laser excitation of the valence electrons, the whole system equilibrates by exchanging energy and momentum, indicated by the double arrows. The 5d and 4f spin systems couple via inter- and intra-atomic exchange, where the intra-atomic exchange *J*_int_=130 meV is much larger than the largest (nearest neighbour) inter-atomic exchange *J*_*ij*_=5.9 meV. In thermal equilibrium, the combination of inter- and intra-atomic exchange interactions mediates spin order in the 4f system via the delocalized 5d valence bands. Upon femtosecond laser excitation, the dynamics of the 5d spin system is dominated by the coupling *α*_e_ to the hot valence electrons described by temperature *T*_e_, while the localized 4f spins couple only to the phonon bath at temperature *T*_p_ via *α*_p_. (**b**) The binding energy versus parallel momentum map *E*_B_(*k*_||_) of Gd recorded with higher-order harmonic radiation (photon energy 36.8 eV) in time- and angle-resolved photoemission gives simultaneously access to the transient exchange splitting of the 5d minority and majority spin bands (↓ and ↑, respectively) and the magnetic linear dichroism of the localized 4f state. Data are plotted on the displayed normalized false colour scale.

**Figure 2:**
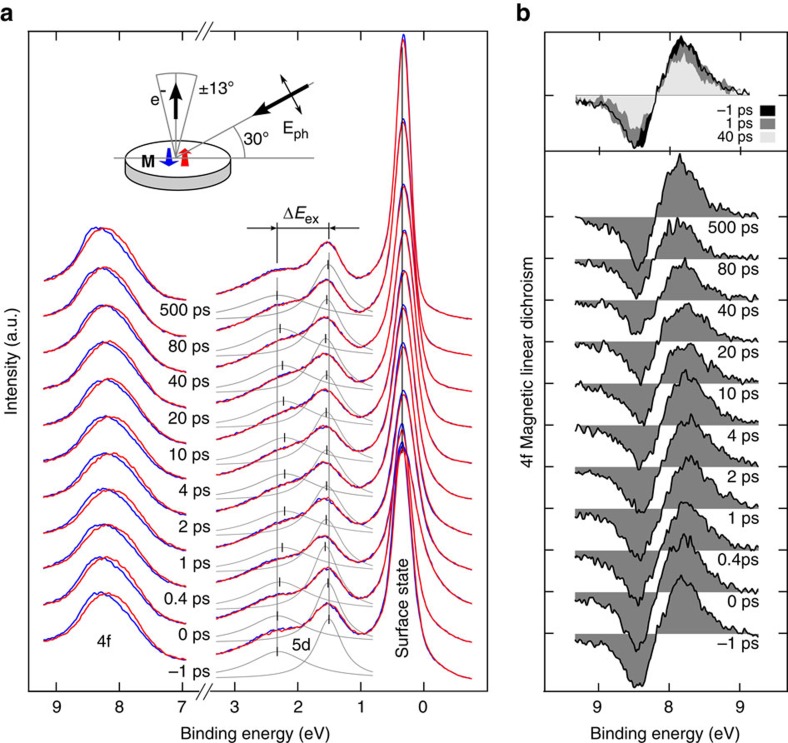
Transient 5d exchange splitting and 4f linear magnetic dichroism of gadolinium. (**a**) The blue and red spectra show the Gd ARPES spectrum as a function of pump–probe delay recorded with p-polarized light in normal emission with a photon energy of 36.8 eV for the two opposite in-plane magnetization directions. Clearly visible are the exchange-split (5d) valence bands at 2 eV binding energy and the 4f core level at 8 eV, in addition to the surface state. The fitted majority and minority spin valence bands are indicated by grey peaks below the actual spectra, the peak positions are marked by black ticks. The inset depicts the experimental geometry for both magnetization directions **M** as indicated by the blue and red arrows. The angle of incidence of the pump and probe beams (bold black arrow) is 30° off the surface plane and the electrons (e^−^) are recorded in Γ–M direction ±13° with respect to the surface normal. The probe beam polarization, *E*_ph_, is indicated by the thin double-headed black arrow. (**b**) The magnetic linear dichroism (MLD) of the 4f electrons is obtained by integrating the absolute value of the difference of two spectra recorded for opposite in-plane magnetizations. Comparison of the signal at three different delays (−1, 1 and 40 ps as indicted by the colour scale) illustrates the slow, picosecond dynamics of the 4f magnetic moment.

To scrutinize the intra-atomic exchange interaction on ultrashort timescales, we probed with orbital resolution the 4f and 5d magnetization dynamics within a single photoemission experiment. We used an apparatus based on high-order harmonic generation^22^ to combine time-resolved ARPES with MLD in the angular distribution of photoelectrons^23^. The sample is a single-crystalline 10-nm-thick Gd(0001) film grown on a tungsten substrate. As shown in Fig. 1b, ARPES with a photon energy of 36.8 eV gives access to the Tamm-like surface state, the transient exchange splitting of the 5d minority and majority spin bands (↓ and ↑, respectively) and the localized 4f state. The Gd 5d electrons and the surface state are excited by the 1.6-eV laser pulse. This photon energy is too low to directly excite the occupied and unoccupied 4f states at 8 eV and −4 eV binding energy^24^. Figure 2a shows a series of photoemission spectra recorded for varying pump–probe delay extracted at the Γ point (averaged over an acceptance window of ±0.1 Å^−1^ in Fig. 1b). As illustrated in the inset, we measured for each delay two photoemission spectra with opposite in-plane magnetization directions (red and blue lines) to determine the 4f MLD.

The occupied majority component of the Tamm-like surface state at binding energy *E*_B_≤0.5 eV indicates the high quality of the Gd film preparation. As the binding energy of the surface state at 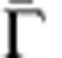 is independent of the magnetization direction, it allows us to correct small space-charge shifts between individual measurements for opposite magnetization directions and equal delay. The small intensity change of the surface state for reversed magnetization is attributed to MLD. We observe a transient shift of the majority surface state towards the Fermi level by ∼50 meV, which is consistent with previous studies^25^,^26^.

**Figure 3:**
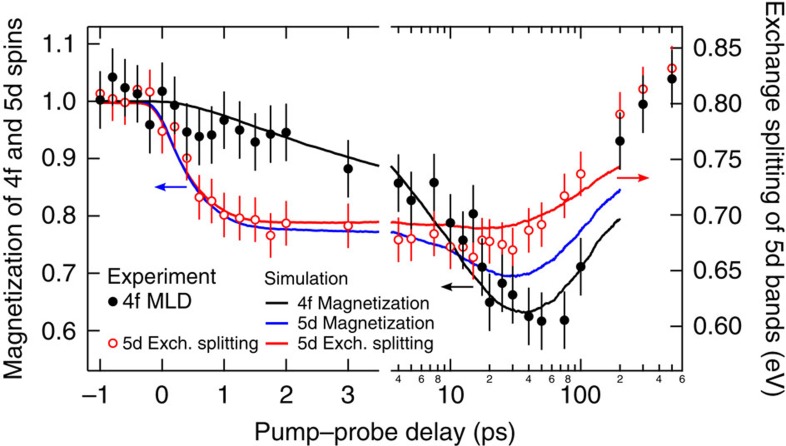
Orbital-resolved spin dynamics in gadolinium. Measured exchange splitting of the 5d bulk bands (red circles, right ordinate) and (normalized) magnetic linear dichroism of the 4f level (black circles, left ordinate) are shown as a function of pump–probe delay recorded with 100-fs XUV pulses. The position of the minority and majority spin valence band was extracted at ±0.1 Å^−1^ around Γ using the fit procedure described in ref. 26. The MLD contrast is evaluated over the 4f photoemission peak with an angular resolution of 0.5° and integrated across the full detection range of ±13° to improve statistics. The error bars show two s.d. The error of the exchange splitting is obtained from the fits of the corresponding bands. The error of the MLD signal is given by the statistical noise of the spectral area at the 4f binding energy. Solid black and blue lines are the (normalized) 4f and 5d magnetic moments calculated with our orbital-resolved spin Hamiltonian. The red solid line is the exchange splitting computed *ab initio* with the calculated 4f and 5d magnetic moments of the spin dynamics simulations as input. Within the first few picoseconds after laser excitation, the dynamics of the exchange splitting and the 5d orbital momentum are synchronous. The decoupling of the intra-atomic exchange is demonstrated by the significantly different demagnetization times of the 5d and 4f spin system. Single exponential fits give time constants of 0.8 and 14 ps, respectively. Note that after 3.5 ps, the dynamics are displayed on a logarithmic scale to cover the cooling back to the initial sample temperature of 90 K.

The exchange-split minority and majority components of the 5d valence band are seen at binding energies of 1.4 and 2.3 eV, respectively. With a probe photon energy of 36.8 eV, we measure the Δ_2_-like component in the 4th Brillouin zone along the Γ–M direction. The valence band dispersion in Fig. 1b agrees well with previous measurements at higher photon energies^27^ and the calculated bulk band structure^28^. The bulk character of these states was also confirmed by recent *ab initio* calculations of a Gd slab^29^. Upon laser excitation, we observe a reduction of the exchange splitting (denoted Δ*E*_ex_ in Fig. 2a), which reaches its minimum within the first picosecond after laser excitation (see also Fig. 3). As substantiated below, the initial drop of the exchange splitting of the valence bands parallels the dynamics of the magnetic moment of the 5d electrons.

To follow in addition the average 4f moment, we simultaneously recorded the intensity contrast of the 4f ^7^*F* final-state spin–orbit multiplet at ∼8 eV binding energy for opposite in-plane magnetization directions. The time evolution of the asymmetry is highlighted in Fig. 2b; the grey area is a measure of the transient 4f magnetic moment^30^. We note that photoemission probes the 5d exchange splitting and 4f MLD in the same sample volume defined by the inelastic mean free path of the photoelectrons of about three monolayers. Although photoemission is a surface-sensitive technique, the MLD contrast reflects mostly the 4f bulk magnetization, that is, the subsurface layers. High-resolution photoemission reveals a surface core-level shift of 0.3 eV in the Gd 4f level with a bulk-to-surface intensity ratio of 3/2 (refs 23, 31). However, the atomic contribution to MLD due to the interference of the d and g photoemission final states is small at our photon energy since the f–g dipole transition is ∼10 times stronger than the f–d counterpart^31^. Thus, the MLD signal mainly originates from photoelectron diffraction where the prevailing forward scattering only enhances the MLD bulk signal. This justifies comparison of the exchange splitting of the 5d valence bands with the 4f MLD. As illustrated in Fig. 2b, we observe a clear reduction in MLD contrast at 40 ps delay, while it is only slightly changed at 1 ps delay (compared with the spectrum recorded before pumping at −1 ps delay).

The normalized exchange splitting and 4f MLD as a function of pump–probe delay are summarized in Fig. 3 by red and black circles, respectively. Their strikingly different temporal evolution reveals ultrafast decoupling of the intra-atomic exchange interaction. While the 5d exchange splitting reaches its minimum after one picosecond, by which time the 5d electron and phonon heat baths are nearly in equilibrium, the 4f magnetization continues to decrease until about 40 ps. The recovery of the magnetization occurs by the time the lattice and spins have comparable temperatures^26^. Fitting the initial collapse of the 5d exchange splitting with a single exponential function yields a time constant of *τ*_5d_=0.8±0.1 ps. This agrees with the time constants from magneto-optical Kerr effect (MOKE) measurements (0.85±0.05 ps)^32^ and our earlier work (0.86±0.1 ps)^26^. For the 4f response, however, we find a much longer single exponential time constant of *τ*_4f_=14±3 ps.

### Orbital-resolved spin dynamics simulations

To shed light on the origin of the disparate magnetization dynamics of the 5d exchange splitting and 4f spin system, we performed atomistic spin dynamics simulations. The 5d moments and the corresponding exchange splitting are caused by the exchange field of the 4f electrons. Nonetheless, it has been shown experimentally^33^ and theoretically^34^ that their behaviour deviates significantly from a Stoner-like behaviour. This motivates us to treat the 4f and 5d moments separately, leading to an orbital-resolved Heisenberg Hamiltonian including the intra-atomic interaction to go beyond the standard model with one fixed spin per atom. This approach was proposed recently and has been shown to describe very well the magnetization switching dynamics in ferrimagnets composed of transition-metal and rare-earth elements^35^; the predicted angular momentum transfer between the 3d and 4f sublattices was confirmed by recent experiments^13^. Moreover, a simple three-temperature model proposed already for the first laser-induced demagnetization experiments on nickel^36^ has been shown to fail for the case of strong demagnetization^37^ or for experiments in Gd^30^, where the spin system itself is driven out of equilibrium^26^. Consequently, Gd requires an orbital-resolved model to describe its two distinct spin systems, as illustrated in Fig. 1a. Here, the 5d spins are coupled to the electronic temperature (*α*_e_) because the 5d electrons are directly excited by the 1.6-eV photons of the pump pulse. In contrast, the 4f electrons are not perturbed by the pump pulse and thus couple, except through intra-atomic 4f–5d exchange *J*_int_, only to the phononic temperature of the system (*α*_p_).

Consequently, we construct the appropriate orbital-dependent spin Hamiltonian as


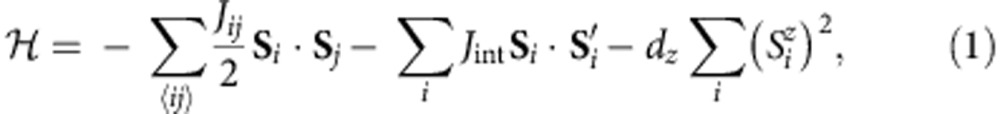


where the 5d and 4f spins are, in the classical limit, expressed by unit vectors **S**_*i*_ and **S′**_*i*_, representing the normalized 5d and 4f magnetic moments, respectively. The first term describes the inter-atomic Heisenberg exchange between the 5d spins at different sites *i*, *j* of the hexagonal close-packed lattice. The second term accounts for the intra-atomic 5d–4f exchange and the third term represents a uniaxial anisotropy. We consider Langevin dynamics, that is, we numerically solve the stochastic Landau–Lifshitz–Gilbert (LLG) equations of motion. For the 5d spins, the LLG equation reads


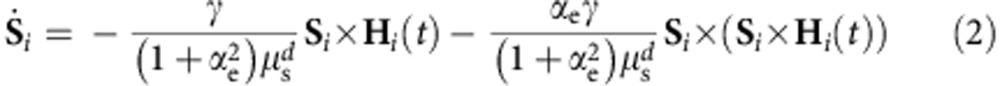


where the phenomenological damping parameter *α*_e_ describes the coupling between 5d spins and the electronic heat bath, 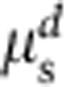 is the 5d spin moment and *γ* denotes the gyromagnetic ratio. The effective field 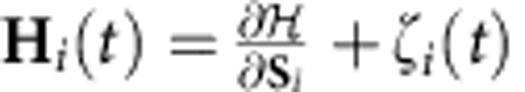 includes thermal fluctuations via the white-noise term *ζ*_*i*_ (ref. 38). The same equation describes the 4f spins ***S***′, however, with a coupling *α*_p_ to the phononic heat bath. Separate values of *α*_e_ and *α*_p_ are not known in the literature, but it turns out that best agreement between simulation and experiment is achieved using different values, namely *α*_e_=0.00013 and *α*_p_=0.0015. Their average is in agreement with the Gilbert damping constant *α*=0.00044 of Gd, known from ferromagnetic resonance^39^. The role of these values is analysed in detail in the Supplementary Discussion and Supplementary Fig. 1.

The exchange constants *J*_*ij*_ and *J*_int_ were calculated *ab initio* using the density functional theory. To validate the exchange constants with our orbital-resolved spin model, we calculated the equilibrium net magnetization and the individual 5d and 4f magnetizations versus temperature (Supplementary Fig. 2). Employing the exchange constants, we simulated a spin system with 45,696 atomic spins, taking into account exchange interactions up to the 22nd nearest neighbour. We computed a Curie temperature (*T*_C_=299 K) close to the experimental value (*T*_C_=293 K), implying that our orbital-dependent *ab initio* exchange constants describe the localized and itinerant magnetism of Gd adequately. Finally, we mention that to compute the electron and phonon temperatures we employed a two-temperature model^40^ using material parameters similar to ref. 41 (see Supplementary Fig. 3 and Supplementary Table 1).

The results of the atomistic spin dynamics simulations for the 5d and 4f moments are shown in Fig. 3 as blue and black solid lines, respectively. Our calculations clearly support a pronounced difference in the demagnetization times of the 4f spins with respect to the 5d spins. To compare directly the exchange splitting predicted by our simulations with its experimental counterpart, we computed the average angle between the 4f and 5d spin moments as a function of pump–probe delay. We then performed *ab initio* calculations for this non-collinear arrangement of the two on-site moments, which gives us the electronic bands, and hence the value of the d-band exchange splitting. The relation between the average angle between the 4f and 5d spin moments and the exchange splitting is given in Supplementary Fig. 4. Note that the 5d exchange splitting computed *ab initio* closely follows the 5d spin moment in the first 10 ps (red and blue curves in Fig. 3, respectively) but deviates more when the 4f moments demagnetize more strongly. This permits us to draw conclusions on the time evolution of the 5d spin moment from the exchange splitting. As can be seen from Fig. 3 the theoretical and measured 5d exchange splitting are in good agreement, as are the theoretical and measured 4f demagnetization. The similarity of measured and simulated 5d and 4f spin moments conclusively proves that despite the massive exchange field, the intra-atomic 5d–4f exchange alignment is broken for tens of picoseconds.

## Discussion

Comparing the measurements with the simulations described above provides the following understanding: laser excitation of the valence electrons in a single-crystalline Gd film creates non-equilibrium conditions between the 5d and 4f spin systems, which persist for several tens of picoseconds. The vastly different energetic positions of the 5d and 4f electrons in Gd are pivotal to the breakdown of the intra-atomic spin alignment on the ultrafast timescale. Initially, only the valence electronic system is heated rapidly by the pump pulse, leading to a fast loss of 5d spin alignment, in spite of the huge exchange field exerted by the spin-polarized 4f electrons. The 4f spin system remains cold for much longer as it couples mainly to the phonon heat bath. Notably, as the 5d electrons reach temperatures of a few thousand Kelvin (see Supplementary Fig. 3), their energy is sufficient to overcome the 4f exchange field. The transient breakdown of 5d–4f intra-atomic alignment only recovers on the slow, picosecond timescale of 4f-spin-lattice relaxation^42^,^43^.

Performing spin dynamics simulations with various *α*_p_ and *α*_e_ damping parameters (see Supplementary Discussion) we find that increasing *α*_p_ ten times does not strongly influence the initial 4f demagnetization, but affects the position of the 4f magnetization minimum at about 70 ps (Fig. 3). Conversely, varying *α*_e_ leads to a stronger change of the initial 5d demagnetization, but does not influence the 5d magnetization minimum at 40 ps. Thus, disparate spin dynamics of 4f and 5d spins are consistently obtained here for a range of damping parameters. A different recent approach^29^ assumes that the 4f and 5d moments in Gd cannot be treated separately and hence predicts the same demagnetization behaviour for 4f and 5d moments, which, however, is not confirmed by our photoemission measurements.

The orbital-resolved spin-dynamics model also has its limitations. Phononic heat transport is neglected, which can cause a somewhat slower cooling in our simulations and thus a magnetization recovery on longer timescales (≥80 ps) than were measured. Note that the difference in the measured and computed recovery times of the 5d and 4f spins is related to the slightly different equilibrium temperature dependence of the 5d and 4f magnetizations (see Supplementary Fig. 2). As mentioned above, our spin-dynamics approach predicts angular momentum transfer between sublattices^35^. For the monoatomic Gd lattice, the transport of 5d spin angular momentum occurs between atoms at differently excited depths of the sample via the inter-atomic exchange coupling. Our model does not include additional spin transport via laser-excited electrons, which we expect to be smaller for Gd than for the 3d ferromagnets due to the smaller net magnetic moment of the Gd 5d valence electrons^44^.

The dynamics of the 4f MLD signal observed in ARPES is in contrast to a previous X-ray magnetic circular dichroism (XMCD) measurement at the Gd M_5_-edge^45^, which probes the unoccupied 4f states. The latter experiment suggests that the demagnetization of the 4f system initially is as fast as that of the directly excited 5d electrons measured with MOKE^46^. The XMCD experiment probes the whole film in transmission, and thus bulk properties. In addition, demagnetization will contain contributions from transport of optically excited electrons generated in the nonmagnetic Y-cap layers^47^ and the Al support into the polycrystalline Gd film. Such additional contributions are not present in our experiment.

Nonetheless, in the photoemission experiment we cannot rule out a small ultrafast drop of the MLD signal followed by a plateau between 0.2 and 2 ps. However, if the 5d and 4f magnetic moments were in equilibrium, the normalized magnetization of the 5d and 4f spin systems would lie on top of each other. Even within the error bars this is clearly not the case. Therefore, the data in Fig. 3 unambiguously support non-equilibrium between the two spin systems lasting for picoseconds. Our photoemission experiment probes the near-surface layers. The 5d and 4f electrons have similar escape depth. In this sample volume, we demonstrate within one measurement disparate spin dynamics, despite the strong intra-atomic exchange. Unravelling the origin of the different 4f dynamics seen in photoemission and X-ray absorption asks for further experimental studies.

The electron and phonon subsystems equilibrate within ∼1.5 ps (see Supplementary Fig. 3). Simultaneous to lattice heating, a strain field will evolve that propagates through the gadolinium film. The impact of such strain fields on the ultrafast magnetization dynamics has recently been discussed for nickel^48^. As crystalline gadolinium has a similar magnetostriction coefficient along its *c* axis^49^, lattice strain may additionally contribute to the demagnetization dynamics. Note that the response of the polycrystalline film probed in XMCD can be quite different, since it is the average of a positive and negative magnetostriction parallel and perpendicular to the *c* axis, respectively^49^. According to the density functional theory calculations^25^, the observed shift of the surface state to lower binding energies by ∼50 meV (Fig. 2 and ref. 26) may point to an expansion of the surface interlayer spacing by about 20 pm. This value is similar to the lattice expansion observed when cooling down Gd bulk from *T*_C_ to about 100 K (refs 49, 50). The anomalous lattice expansion of Gd is related to magnetostriction and indicates a repulsion between the ferromagnetic layers. Vice versa, expansion of the lattice upon laser excitation starting at the surface may stabilize the ferromagnetic state. These arguments are, however, challenged by the ultrafast (≤50 fs) and significant (≥50%) drop of the surface-sensitive magnetic second harmonic signal^25^, which indicates ultrafast demagnetization of the Gd surface layer^41^, as well as by the comparable dynamics of 5d exchange splitting and MOKE^32^. These techniques probe near-surface layers and bulk, respectively.

Our orbital-resolved spin dynamics simulations show that despite the strong intra-atomic exchange, disparate transient spin dynamics can occur. Recently, transient decouplings have been observed for the inter-atomic exchange in permalloy, which showed a ∼20-fs shift in the transversal-MOKE response between the Fe and Ni M-edges^9^, as well as in GdFeCo alloy, where the inter-atomic exchange is much weaker (≲3 meV) and the Gd–Fe decoupling lasted a few picosecond^8^. Here we show for the first time not only decoupling of the much stronger intra-atomic exchange (*J*_int_=130 meV), but also that this decoupling lasts for about 40 ps. We note further that the intra-atomic exchange interaction has been considered previously in the context of laser-induced magnetization dynamics in ferromagnetic semiconductors^51^ and in laser-induced phase transitions in manganites^7^. In the former study, the influence of exchange coupling between localized and itinerant spin degrees of freedom was evaluated. In the latter study, quantum spin-flips mediated by the Hund's rule coupling of Mn 3d states were proposed for fast switching of the magnetic order. In our approach, we extend in a different way beyond the classical spin limit of one spin per atom, by introducing exchange coupled, orbital-specific spins on an individual Gd atom. As confirmed by recent experiments^13^ and the presented simulations, our approach provides indeed a very good explanation of the orbital-selective spin dynamics. Since the large energy separation of the 5d and 4f electrons is specific to Gd, a similar observation of transient decoupling in other lanthanide metals will be difficult, as laser irradiation can rapidly heat both 4f and 5d systems.

In conclusion, femtosecond laser-pulse excitation allows us to drive the 5d and 4f spin systems of gadolinium metal out of equilibrium. Despite the strong intra-atomic exchange interaction, their demagnetization dynamics is characterized by time constants that differ by one order of magnitude. Their surprisingly disparate time evolution is well explained by orbital-resolved spin dynamics simulations based on exchange parameters calculated *ab initio*. Our simultaneous examination of the localized and itinerant magnetism in Gd evidences that ultrafast laser stimulation of the valence electrons offers a route to transiently overcome the massive 4f–5d exchange interaction. Understanding thereby the operation of fundamental magnetic interactions at ultrashort timescales and realizing the ability to manipulate them may have tremendous implications for future magnetic storage devices.

## Methods

### Molecular beam epitaxy of Gd films on W(110)

Single-crystalline, 10-nm-thick Gd(0001) films were grown epitaxially on a W(110) crystal at room temperature. The pressure during evaporation was 10^−10^ mbar. Subsequent annealing to 700 K allows the Gd lattice to relax and improves the film quality. Film thickness was calibrated by a quartz microbalance, film cleanliness and order were verified by low-energy electron diffraction and photoemission spectroscopy. The surface state intensity is a sensitive probe of the sample surface quality (see Fig. 2a and Supplementary Fig. 5).

### Time- and angle-resolved photoemission spectroscopy

Pump and probe pulses for the time-resolved ARPES experiment were derived from a femtosecond Ti:saphire chirped-pulse laser amplifier. The laser was running at 10 kHz, producing broadband pulses at a centre wavelength of 775 nm. A beam splitter transmits 200-μJ pump pulses, which were incompletely compressed to 300 fs duration in a separate compressor and adjusted in power and focus size. After compression to 45 fs, the remaining 1.3 mJ were focused into 100 mbar argon to produce XUV probe pulses. A toroidal grating monochromator selected the 23rd harmonics with a photon energy of 36.8 eV. For the experiment we used p-polarized probe pulses with a duration of 100 fs at a bandwidth of 150 meV. The pump pulse has a photon energy of 1.6 eV and was stretched to 300 fs pulse duration to reduce space-charge effects. The absorbed fluence is 3.5±1 mJ cm^−2^.

The ARPES experiments were conducted at 3 × 10^−11^ mbar with a view-type 100-mm hemispherical photoelectron analyzer. The exchange splitting of the 5d bands was derived from energy distribution curves at normal emission (integrated at ±0.1 Å^−1^ in Γ–M direction). The surface state, as well as minority and majority spin components of the 5d band were fitted using Lorentzian line shapes (see Supplementary Fig. 5).

For the MLD signal, we corrected slight differences in the space-charge shift between the two magnetization directions by aligning the spectra at the surface state. This is appropriate because the surface state has vanishing Rashba splitting at the Γ-point^31^. The electron distribution curves for both magnetization directions were normalized in intensity before subtracting one from the other for each pump–probe delay. The MLD signal is the integral over the absolute value of the intensity difference of the 4f state for opposite in-plane magnetization directions. In thermal equilibrium this value is proportional to the 4f magnetization of the sample^30^,^52^.

### Density functional theory calculations

For the calculation of the intra- and inter-atomic exchange constants, we have adopted two different computational schemes. The intra-atomic exchange constant *J*_int_=130 meV was calculated with the full-potential linear augmented plane wave method within the local spin density approximation (LSDA), employing the band-structure program ELK. Here, the 4f electrons were included in the valence states. This is necessary for describing correctly the interaction between the 5d and 4f states. The program was modified to allow constraining the magnetizations of the spd and 4f states into an antiparallel alignment (see ref. 35 for details). The computed intra-atomic exchange constant is in good agreement with a previous calculation^34^.

For the *ab initio* calculation of the inter-atomic exchange constants, we have found the approach in which the 4f electrons are treated as a part of the core states to be the most efficient. The self-consistent electronic structure was calculated using the tight-binding linear muffin-tin orbital method^53^, adopting the LSDA^54^. Treating the f electrons as localized core electrons notably helps to overcome some of the inaccuracies of the LSDA when applied to Gd, namely, its prediction of an antiferromagnetic ground state^55^ related to the positioning of the minority spin 4f states too close to the Fermi level^28^. Also, this approach has been successfully applied to predict the spontaneous volume magnetostriction in Gd^34^.

For the calculation of the inter-atomic exchange constants *J*_*ij*_ (≤5.9 meV), we have employed the mapping of the magnetic behaviour of the real material onto an effective Heisenberg Hamiltonian^56^,^57^. Specifically, we have used the magnetic force theorem approach^56^, which allows infinitesimal changes of the total energy to be expressed in terms of the one-particle eigenvalues containing the non-self-consistent changes of the effective one-electron potential accompanying the infinitesimal rotations of the spin quantization axes, that is, without any additional self-consistent calculations besides that for the collinear ground state. The resulting pair-exchange constants are given by


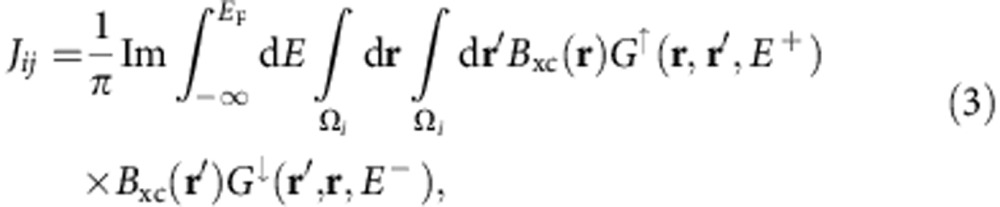


where *E*_F_ denotes the Fermi level, Ω_*i*_ denotes the i-th atomic cell, *σ*=↑,↓ is the spin index, *E*^+^=lim_*α*→0_*E*+i*α*, *G*^*σ*^ are spin-dependent one-electron retarded Green functions and *B*_xc_ is the magnetic field from the exchange-correlation potential. The validity of this approximation has been examined quantitatively in recent studies and it was found to be rather successful in explaining the thermodynamic properties of a broad class of magnetic materials^58^,^59^.

The exchange constants *J*_*ij*_ have been computed up to the 29th nearest-neighbour shell. We have used around a million *k*-points in the full Brillouin zone for energy points close to the Fermi level. In both tight-binding linear muffin-tin orbital and full-potential linear augmented plane wave calculations, the Gd lattice constant adopted was 3.629 Å and the *c*/*a* ratio, 1.597.

### Modelling of electronic and lattice temperature

To model the electronic and lattice temperature, we use the well-established two-temperature model^40^,^60^. Thereby perpendicular heat diffusion in the electronic sub-system is included. Furthermore, the energy flow into the spin system is taken into account by numerically calculating the time derivative of the Hamiltonian in equation (1) at every time step and adding it to the two-temperature model (see Supplementary Note).

## Additional information

**How to cite this article**: Frietsch, B. *et al.* Disparate ultrafast dynamics of itinerant and localized magnetic moments in gadolinium metal. *Nat. Commun.* 6:8262 doi: 10.1038/ncomms9262 (2015).

## Supplementary Material

Supplementary Information Supplementary Figures 1-5, Supplementary Table 1, Supplementary Note, Supplementary Discussion, and Supplementary References.
