# Supplementary material for: Disparate ultrafast dynamics of itinerant and localized magnetic moments in gadolinium metal
Source: Nat Commun. 2015 Sep 10;6:8262. doi: 10.1038/ncomms9262 (PMC4579838; doi:10.1038/ncomms9262)
Supplement: Supplementary Information — Supplementary Figures 1-5, Supplementary Table 1, Supplementary Note, Supplementary Discussion, and Supplementary References. [file ncomms9262-s1.pdf]

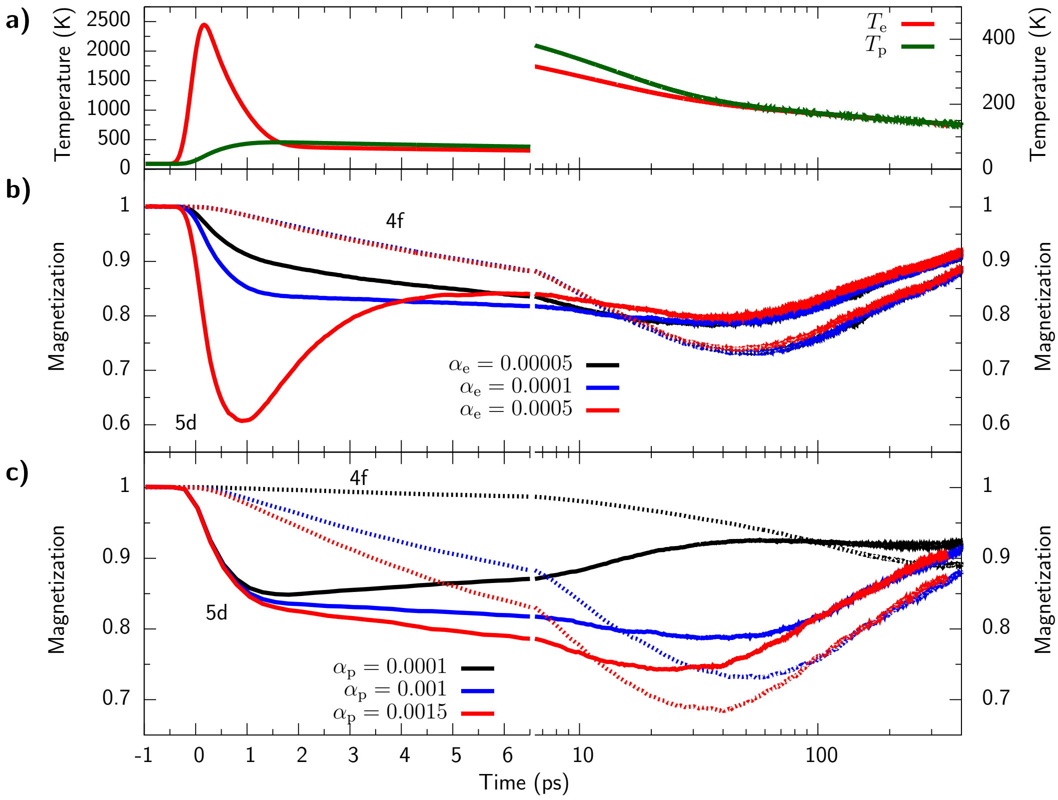

Supplementary Figure 1: **Role of damping constants on the magnetization dynamics. a)** Time evolutions of the electron and phonon temperatures T_e and T_p of the laser excited 40 nm Gd film on tungsten, calculated with the improved temperature model (see Section Supplementary Notes). Influence of the values of the damping parameters **b)** α_e and **c)** α_p on the computed magnetization dynamics (see Section Supplementary Discussion). The value of α_e affects mainly the degree of demagnetization of the 5d orbitals on a time scale of few picoseconds. The value of α_p concerns mostly the magnetization dynamics on longer time scales. The blue data sets in panels b) and c) are identical ($\alpha_e = 0.0001$, $\alpha_p = 0.001$).

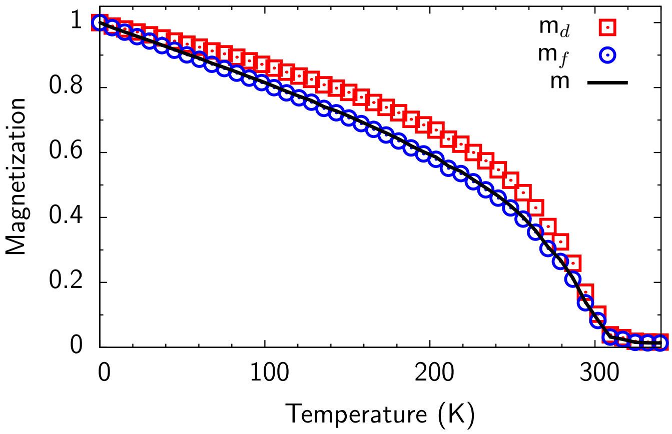

Supplementary Figure 2: **Temperature-dependence of the equilibrium magnetization of gadolinium.** Equilibrium normalized net magnetization m and individual magnetizations of the 5d and 4f spin system m_d and m_f versus temperature calculated with the orbital-resolved spin Hamiltonian by numerically solving the stochastic Landau-Lifshitz-Gilbert equations (see main text). All exchange constants were derived from density-functional theory calculations. The calculated Curie temperature of $T_\mathrm{C} = 299$ K agrees well with the experimental value of 293 K.

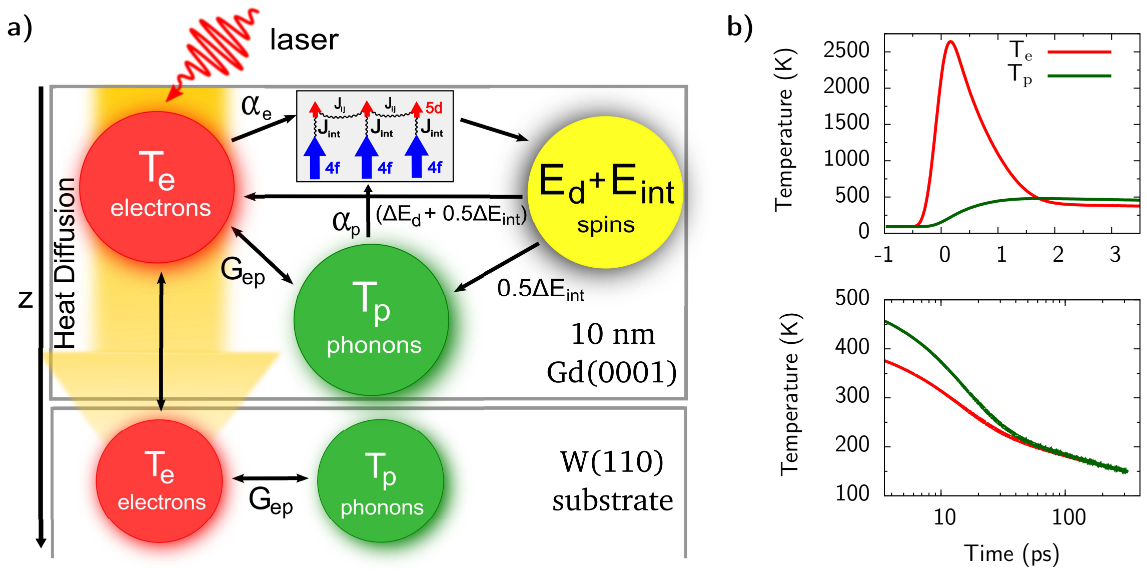

Supplementary Figure 3: **Electronic and lattice temperatures.** (a) Sketch of the improved temperature model. The laser pulse excites the electronic systems of the Gd film and the W substrate. Due to electron-phonon scattering the electron and phonon sub-systems equilibrate. Heat diffusion in the electronic sub-system leads to additional cooling of the sample. Via the damping constants α_e and α_p the electronic and phononic heat baths are coupled to the spin system. The calculated energy flow into the spin system leads to an additional cooling of these heat baths. (b) Calculated temperature evolution of the first Gd layer after laser excitation with a 300-fs pulse for an absorbed pump fluence of $4\,mJ/cm^2$.

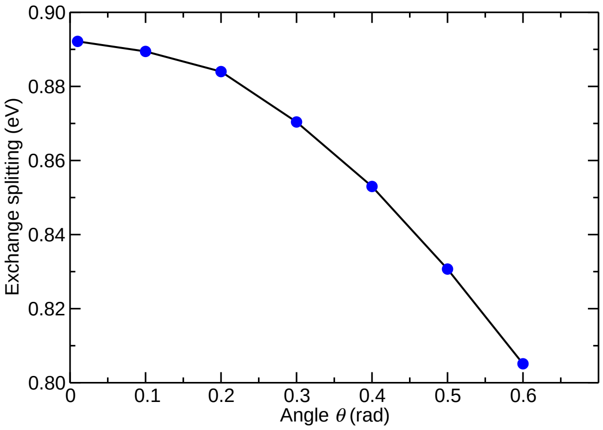

Supplementary Figure 4: **Exchange splitting for non-collinear arrangement of 5d and 4f moments.** *Ab initio* computed d-band exchange splitting at the Γ point as a function of the angle θ between the non-collinear 5d and 4f spin moments on Gd. The spin-dynamics simulations give for every time step t a 5d and a 4f spin moment on each Gd atom and the average angle θ between these 4f and 5d spin moments. Using the computed values of θ we perform *ab initio* calculations for this non-collinear arrangement of the two on-site moments, which gives us the electronic bands and hence the value of the d-band exchange splitting for every non-collinear arrangement.

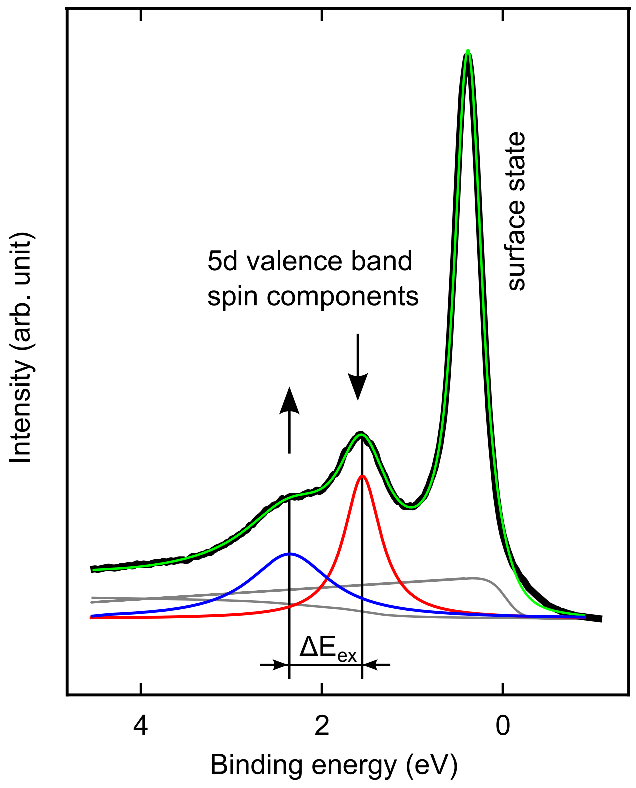

Supplementary Figure 5: **Energy distribution curve showing the gadolinium valence band structure at the Γ-point.** Exemplary fit of an energy distribution curve recorded at negative pump-probe delays. The spectrum is dominated by the majority spin surface state close below the Fermi level. Majority (blue, ↑) and minority (red, ↓) spin components of the 5d valence band show a clear exchange splitting ΔE_{ex}. The surface state, as well as the minority and majority spin components of the 5d band were fitted using Lorentzian line shapes. A Shirley background is added to describe the inelastic scattering of photoelectrons, along with a linear background that accounts for elastic electron scattering from other points in the Brillouin zone. The spectrum was convoluted with a 150 meV Gaussian function to account for the instrumental resolution resulting in the thin green line. As expected the two reversed magnetization directions showed the same dynamics in the 5d exchange splitting. We used the sum of both to achieve better statistics.

	Gadolinium	Tungsten
$C_\mathrm{e} = \gamma T_\mathrm{e}$	$225 \frac{\mathrm{J}}{\mathrm{m^3K^2}} \cdot T_\mathrm{e}$ [1]	$138 \frac{\mathrm{J}}{\mathrm{m^3K^2}} \cdot T_\mathrm{e}$ [2]
C_p	Debye Model	$2.6 \cdot 10^6 \frac{\mathrm{J}}{\mathrm{m^3K}}$
G_ep	$2.5 \cdot 10^{17} \frac{\mathrm{W}}{\mathrm{m^3K}}$	$2 \cdot 10^{17} \frac{\mathrm{W}}{\mathrm{m^3K}}$ [2]
κ_0	$11 \frac{\mathrm{W}}{\mathrm{mK}}$ [3]	$173 \frac{\mathrm{W}}{\mathrm{mK}}$
δ	40 nm	23.6 nm

Supplementary Table 1: Values of the constants used in the improved two temperature model for a Gd film on W(110).

Supplementary Notes

The temperature evolution in metals following excitation with a fs laser pulse is well described by the established two-temperature model (TTM) derived by Kaganov *et al.* [4]. We use specifically the form of Anisimov *et al.* [5], where perpendicular heat diffusion in the electronic subsystem is taken into account. Due to the large electronic heat conductivity of the tungsten substrate [2], heat transport plays a significant role, even on very short timescales. Within the TTM approach the magnetic subsystem is usually neglected. For Gd it is known that the 4f spin-system contributes significantly to the heat capacity [6] and has therefore to be taken into account. In former publications this has been done by simply adding its equilibrium contribution to the phonon heat capacity [7–9]. Since the spin systems are far from equilibrium on a picosecond time scale this would overestimate their contribution. Therefore we calculate the energy flow into the spin system directly by numerically calculating the time-derivative of the Hamiltonian (Eq. (1) in the main text) at every time step and adding it to the TTM. Supplementary Figure **3**(a) sketches this improved model for the temperature evolution of the electron and lattice subsystem.

The two coupled differential equations for our temperature model then read as follows:

$$C_\mathrm{e}\frac{\partial T_\mathrm{e}}{\partial t} = G_\mathrm{ep}(T_\mathrm{p}-T_\mathrm{e}) + P(z,t) + \frac{\partial}{\partial z}\kappa\frac{\partial T_\mathrm{e}}{\partial z} - \frac{\partial(E_\mathrm{d}+0.5E_\mathrm{int})}{\partial t}, \quad (1)$$

$$C_\mathrm{p}\frac{\partial T_\mathrm{p}}{\partial t} = G_\mathrm{ep}(T_\mathrm{e}-T_\mathrm{p}) - 0.5\frac{\partial E_\mathrm{int}}{\partial t}. \quad (2)$$

Both the electron temperature T_e as well as the phonon temperature T_p depend on the layer depth z. $C_\mathrm{e} = \gamma T_\mathrm{e}$ and C_p are the electron and lattice specific heat capacities. While the heat capacity for W is assumed to be constant, we use the Debye approximation with a Debye temperature of $\Theta_\mathrm{D} = 163\,\mathrm{K}$ for Gd [10]. The first term on the right hand side of both equations describes the electron-phonon coupling as derived by Kaganov *et al.* [4]. $P(z,t)$ describes the absorbed energy in the different layers, where we assume an exponential decay from the surface of the sample. We further follow Hohlfeld *et al.* [11] and use an increased effective penetration depth of $\delta_\mathrm{Gd} = 40$ nm in Gd to include ballistic transport. The third term in the first equation describes heat diffusion in the electronic subsystem. Here, $\kappa = \kappa_0 T_\mathrm{e}/T_\mathrm{p}$ is the thermal heat conductivity. The last term in both equations describes the energy flow into the spin system. Here, E_d is the energy density due to the inter-atomic exchange and the anisotropy of the 5d spin system and E_int is the

energy density due to the intra-atomic exchange. Since the hot electron system leads to a fast energy transfer into the 5d spin system, we add this energy flow to the equation for the electron temperature. On the other hand the 4f spin system is only coupled to the phonon temperature bath, so we add half of the energy flow into the intra-atomic exchange to the equation for the phonon temperature. Supplementary Figure **3**(b) shows the temperature evolution after excitation with a 300-fs laser pulse with an absorbed fluence of 4 mJ/cm^2 as used for the simulation in the manuscript. Supplementary Table **1** lists the material constants used in the numerical simulation of the improved two temperature model.

Supplementary Discussion

Our spin-dynamics simulations are influenced by the choice of the coupling constants α_{e} and α_{p} describing the heat flow between the spin system and the electronic and phononic heat baths. They are the only unknown parameters in our simulation with no reason to assume equal values. However, the more macroscopic Gilbert damping parameter has been measured to be $\alpha = 0.0004$ in Gd — a rather low value which should manifest itself in our microscopic coupling parameters. Supplementary Figure **1** demonstrates the influence of the values of these parameters on the magnetization dynamics. A variation of α_{e} affects mainly the degree of demagnetization of the 5d orbitals on very short time scales where the high electron temperature plays the dominant role. The value of α_{p} concerns mostly the magnetization dynamics on longer time scales, where — due to the strong intra-atomic exchange — the magnetization dynamics of both d and f orbitals is affected. The best agreement with the experiment is achieved for values of $\alpha_{\mathrm{e}} = 0.00013$ and $\alpha_{\mathrm{p}} = 0.0015$. Simulations with these values are shown in the main part of the manuscript. Note, that our main result — the distinct dynamics of the 5d and 4f spin moments — is not affected by the choice of these parameters as long as they are not orders of magnitude larger than the measured macroscopic Gilbert damping.

Supplementary References

[1] Hill, R. W., Collocott, S. J., Gschneidner, Jr., K. A. & Schmidt, F. A. The heat capacity of high-purity gadolinium from 0.5 to 4 K and the effects of interstitial impurities. *J. Phys. F: Metal Phys.* **17**, 1867–1884 (1987).

[2] Lin, Z., Zhigilei, L. V. & Celli, V. Electron-phonon coupling and electron heat capacity of metals under conditions of strong electron-phonon nonequilibrium. *Phys. Rev. B* **77**, 075133 (2008).

[3] Jacobsson, P. & Sundqvist, B. Thermal conductivity and electrical resistivity of gadolinium as functions of pressure and temperature. *Phys. Rev. B* **40**, 9541–9551 (1989).

[4] Kaganov, M. I., Lifshitz, I. M. & Tanatarov, L. V. Relaxation between electrons and the crystalline lattice. *Sov. Phys. JETP* **4**, 173–178 (1957).

[5] Anisimov, S. I., Kapeliovich, B. L. & Perelman, T. L. Electron emission from metal surfaces exposed to ultrashort laser pulses. *Sov. Phys. JETP* **39**, 375–377 (1974).

[6] Jelinek, F. J., Gerstein, B. C., Griffel, M., Skochdopole, R. E. & Spedding, F. H. Re-evaluation of some thermodynamic properties of gadolinium metal. *Phys. Rev.* **149**, 489–490 (1966).

[7] Bovensiepen, U. Coherent and incoherent excitations of the Gd(0001) surface on ultrafast timescales. *J. Phys.: Condens. Matter* **19**, 083201 (2007).

[8] Melnikov, A. *et al.* Nonequilibrium magnetization dynamics of gadolinium studied by magnetic linear dichroism in time-resolved $4f$ core-level photoemission. *Phys. Rev. Lett.* **100**, 107202 (2008).

[9] Sultan, M., Atxitia, U., Melnikov, A., Chubykalo-Fesenko, O. & Bovensiepen, U. Electron- and phonon-mediated ultrafast magnetization dynamics of Gd(0001). *Phys. Rev. B* **85**, 184407 (2012).

[10] Tsang, T.-W. E., Gschneidner, Jr., K. A., Schmidt, F. A. & Thome, D. K. Low-temperature heat capacity of electrotransport-purified scandium, yttrium, gadolinium, and lutetium. *Phys. Rev. B* **31**, 235–244 (1985).

[11] Hohlfeld, J. *et al.* Electron and lattice dynamics following optical excitation of metals. *Chem. Phys.* **251**, 237–258 (2000).
